# IgG_4_ antibodies to the recombinant filarial antigen Wb-Bhp-1 decrease dramatically following treatment of lymphatic filariasis

**DOI:** 10.1371/journal.pntd.0011364

**Published:** 2023-06-07

**Authors:** Sarah E. Greene, Yuefang Huang, Kurt C. Curtis, Christopher L. King, Peter U. Fischer, Gary J. Weil

**Affiliations:** 1 Infectious Diseases Division, Department of Pediatrics, Washington University School of Medicine, St Louis, Missouri, United States of America; 2 Infectious Diseases Division, Department of Medicine, Washington University School of Medicine, St Louis, Missouri, United States of America; 3 Center for Global Health and Diseases, Case Western Reserve University, Cleveland, Ohio, United States of America; 4 Veteran Affairs Medical Center, Cleveland, Ohio, United States of America; Universidade Federal de Minas Gerais, BRAZIL

## Abstract

**Background:**

Lymphatic filariasis (LF) is a neglected tropical disease and a major cause of chronic disability. Improved diagnostic tests are needed because of long-term persistence of anti-filarial antibodies or circulating filarial antigenemia after treatments that clear microfilaremia. Here, we assess changes in levels of antibodies to the recombinant filarial antigens Wb-Bhp-1, Wb123, and Bm14 after anti-filarial treatment.

**Methodology/principal findings:**

IgG_4_ antibodies to recombinant filarial antigens were assessed by ELISA. We tested serial plasma samples from a clinical trial in Papua New Guinea. Before treatment, 90%, 71% and 99% of participants had antibodies to Wb-Bhp-1, Wb123, and Bm14, respectively. Antibodies to Wb-Bhp-1 and Wb123, but not Bm14, were significantly higher in participants with persistent microfilaremia 24 months after treatment. Antibodies to all three antigens declined significantly by 60 months after treatment with ivermectin, diethylcarbamazine and albendazole despite circulating filarial antigen in 76% of participants. By 60 months follow up, antibodies to Wb-Bhp-1, Wb123, and Bm14 were detected in 17%, 7% and 90% of participants, respectively. Antibodies to Wb-Bhp-1 also declined more rapidly after treatment than antibodies to Bm14 in samples from a clinical trial conducted in Sri Lanka. We also tested archived serum samples from people living in filariasis-endemic communities in Egypt with different infection profiles. Antibodies to Wb-Bhp-1 were detected in 73% of microfilaremic people, 53% of amicrofilaremic people with circulating filarial antigen, and 17.5% of endemic individuals without microfilaria or circulating filarial antigen. Tests performed with legacy samples from India showed that few people with filarial lymphedema had antibodies to these recombinant antigens.

**Conclusions:**

Antibodies to Wb-Bhp-1 and Wb123 are more closely correlated with persistent microfilaremia than circulating filarial antigenemia or antibodies to Bm14, and they clear more rapidly after anti-filarial treatment. Additional studies are needed to assess the value of Wb-Bhp-1 serology as a tool for determining the success of LF elimination efforts.

## Author summary

Lymphatic filariasis (LF) is a neglected tropical disease targeted for elimination by the World Health Organization. Public health programs aim to eliminate the infection with repeated rounds of mass distribution of anti-filarial medicines in areas with LF. These mass drug administration campaigns have been highly successful. Improved diagnostic tests are needed to monitor and verify the success of infection elimination efforts. We have previously shown that detection of antibodies to the recombinant parasite protein Wb-Bhp-1 are sensitive and specific for diagnosis of active filarial infections. Here, we show that antibodies to Wb-Bhp-1 are correlated with the persistence of filarial parasites in the blood after treatment, and that they decrease after effective anti-filarial treatment. Importantly, antibodies to Wb-Bhp-1 decrease after treatment more rapidly than other diagnostic markers such as circulating filarial antigenemia or antibodies to Bm14. Thus, this antibody test may be useful as a tool for monitoring the success of filariasis elimination programs.

## Introduction

Lymphatic filariasis (LF) is a neglected tropical disease caused by closely related parasitic nematodes, most commonly *Wuchereria bancrofti*, *Brugia malayi* and *B*. *timori*. Mosquitoes transmit the infectious stage of the filarial parasites to humans. Adult filarial worms live in lymphatic vessels where they cause lymphatic damage that can lead to disabling lymphedema and hydroceles. Significant progress has been made in controlling and eliminating this infection by the World Health Organization’s (WHO) Global Programme to Eliminate LF (GPELF). The GPELF uses mass drug administration (MDA) as large scale preventative chemotherapy to treat infections and interrupt transmission. The combination of ivermectin, diethylcarbamazine (DEC) and albendazole, IDA, is more effective at clearing microfilaria (Mf) than albendazole with either DEC or Ivermectin [1–3]. Current GPELF protocols call for continuing MDA if circulating filarial antigenemia (CFA) prevalence exceeds 2% [4]. However, CFA prevalence often remains >2% long after Mf prevalence is well below 1% [5]. Diagnostic tools that correlate more closely with Mf could be especially helpful for informing MDA stopping decisions. Indeed, the WHO cites the development of improved diagnostics as an important priority for the LF elimination program [6,7].

There are several commercially available antibody tests for LF including those that detect antibodies to the recombinant proteins Bm14 (rBM14), Wb123 (rWb123) and BmR1 (rBmR1) [8–12]. Antibody based diagnostics are desirable as anti-filarial antibodies can be detected sooner after infection than Mf or circulating filarial antigen (CFA); thus, antibody tests may be a more sensitive method for detecting early infections in children [13,14]. A major limitation of antibody-based diagnostic tests is prolonged persistence of antibodies after treatment successfully clears microfilariae from the blood. This has been seen with several different anti-filarial antibody tests, although there has been variability in the reported duration of anti-filarial antibody positivity after treatment [10,15–18].

We have previously identified and characterized an ELISA test that detects antibodies to recombinant Wb-Bhp-1 (rWb-Bhp-1), a *W*. *bancrofti* homologue of BmR1, the antigen target of antibodies detected in the Brugia Rapid test [19]. Here, we set out to further evaluate this test with samples collected before and after anti-filarial treatment. We compared results obtained with this test to those obtained with other tests, such as Mf detection, CFA, and other antibody tests. We found that antibodies to rWb-Bhp-1 clear more rapidly after treatment. Therefore, they may be a better marker for Mf persistence after treatment than CFA or antibodies to Bm14.

## Methods

### Ethics statement

Samples from the clinical trial in Papua New Guinea were obtained after approval by the institutional review board at University Hospital Cleveland Medical Center and the Papua New Guinea Institute of Medical Research and Medical Research Advisory committee, and after written informed consent was obtained from each participant. These samples were deidentified and sent to Washington University for antibody testing. All other clinical samples were de-identified legacy samples and linked to metadata regarding infection status and treatment history by study identification numbers only. The Washington University in St Louis Human Research Protection Office (an institutional review board) determined that work with these de-identified legacy samples did not constitute human subjects research.

### Serum and plasma samples

All serum and plasma samples used in this study were deidentified legacy samples collected from people with documented *W*. *bancrofti* infection, clinically evident LF, or who resided in a *W*. *bancrofti* endemic area, as detailed in Table 1.

**Table 1:** Description of serum or plasma samples used in this study.

Location of collection	Number of samples	Infection status	Citation
**Egypt**	42	*W*. *bancrofti* (Mf and/or CFA positive, as described in the text)	[20]
	40	Uninfected (Mf and CFA negative)	[20]
**India**	16	People with filarial lymphedema or hydrocele (+/- Mf, as described in the text)	[21]
**Papua New Guinea**	97	*W*. *bancrofti* (Mf positive before treatment)	[2]
	99	*W*. *bancrofti* (24 months after treatment)	[2]
	71	*W*. *bancrofti* (36 months after treatment)	[2]
	29	*W*. *bancrofti* (60 months after treatment)	[1,2]
**Sri Lanka**	24	*W*. *bancrofti* (Mf positive before treatment)	[22]
	24	*W*. *bancrofti* (24 months after treatment)	[22]
**Cote d’Ivoire**	5	*W*. *bancrofti* (Mf positive)	[3]

### Detection of anti-filarial antibodies by indirect ELISA

Recombinant filarial antigens rWb-Bhp-1 [19], and rBm14 [23], were produced as previously described. rWb123 [10] was provided as a gift from Dr. Thomas Nutman (National Institute of Health, Bethesda, Md). 96 well polyvinyl round bottom plates were coated with 100ul of antigen diluted to the following concentrations in 0.06M carbonate buffer pH 9.6: 0.5 μg/ml rWb-Bhp-1, 5 μg/ml rWb123, or 2 μg/ml rBm14. Plates were covered and incubated at 37°C overnight in a humidified chamber. Plates were washed twice in PBS-Tween (PBST), and then blocked with PBST with 5% heat inactivated fetal calf sera (ELISA diluent) at 37°C for 1 hour (hr). Human serum or plasma diluted at 1:100 in ELISA diluent was added (100ul per well in duplicate) and plates were incubated at 37°C for 2 hr. Plates were washed 5 times with PBST before addition of 100ul/well of anti-human IgG_4_-pFc’-conjugated to horseradish peroxidase (HRP) (Southern Biotech, Birmingham, AL, USA) diluted at 1:4000 in ELISA diluent, and plates were incubated at 37°C for 1 hr. Plates were washed in PBST 5 times before adding the substrate o-phenylenediamine dihydrochloride (Thermo Fisher Scientific, Waltham MA, USA). The enzymatic reaction was stopped with 4M H_2_SO_4_ and plates were read at 490 nm with a BioTek ELx808 plate reader (Thermo Fisher Scientific). Optical density values from duplicate wells were averaged. A positive cutoff of OD_490_ > 0.2 was used to maximize sensitivity and specificity in the anti-rWb-Bhp-1 ELISA, as previously reported [19]. The same positive cutoff was selected for the anti-rBm14 and anti-rWb123 ELISA for consistency. This is similar to previously reported cutoffs for assays with Bm14 [16].

### Circulating filarial antigen ELISA

This antigen capture immunoassay was performed as previously described [24]. Briefly, human sera were boiled with EDTA to release antigen from immune complexes. Polyvinyl plates were coated with monoclonal DH6.5 then prepared sera or antigen control were added to duplicate wells. After 2 hours, plates were washed and peroxidase conjugated AD12.1 was added. Plates were washed again then exposed with OPD and the plates are read at OD_490_.

### Generation of polyclonal antibodies to rWb-Bhp-1

We utilized mouse anti-rWb-Bhp-1 that was prepared as previously described [19]. We also developed rabbit polyclonal anti-rWb-Bhp-1 antibodies that were produced in 2 rabbits each given 2 doses of rWb-Bhp-1. The polyclonal rabbit anti-rWb-Bhp-1 were then affinity purified. These antibodies were produced using rWb-Bhp-1 we supplied (LifeTein, Somerset NJ, USA). Both antibodies recognize rWb-Bhp-1 in immunoblots. Epitope mapping was not performed, but we presume that these antibodies can bind to different epitopes given that they can be used in conjunction in a sandwich ELISA (described below).

### Sandwich ELISA to detect Wb-Bhp-1 in human plasma

96 well polyvinyl round bottom plates were coated with 20 μg/ml of affinity purified rabbit anti-rWb-Bhp-1 polyclonal antibody in 0.1M sodium carbonate buffer pH 8, incubated overnight at 37°C, then blocked with ELISA diluent at 37°C for 1 hour. Plates were coated with sera that had been boiled 5 min with an equal volume 0.1M EDTA pH 7.5 or with 500 μg/ml purified rWb-Bhp-1 boiled in 0.1M EDTA pH 7.5, an antigen that we have shown is heat stable. Plates were washed 5 times with PBST, incubated at 37°C for 1 hour with 1:1000 dilution of mouse anti-rWb-Bhp-1. Plates were washed 5 times with PBST, before HRP conjugated anti-mouse IgG (Southern Biotech) diluted at 1:4000 in ELISA diluent was added, and plates were incubated at 37°C for 1 hr. Plates were washed in PBST 5 times before adding the substrate o-phenylenediamine dihydrochloride. The enzymatic reaction was stopped with 4M H_2_SO_4_, and plates were read as described above.

### Statistical analysis

Statistical analyses were conducted in Excel (Microsoft, Redmond WA, USA) and Prism Version 9 (GraphPad).

## Results

### Changes in anti-filarial antibodies after treatment

**Fig 1:**
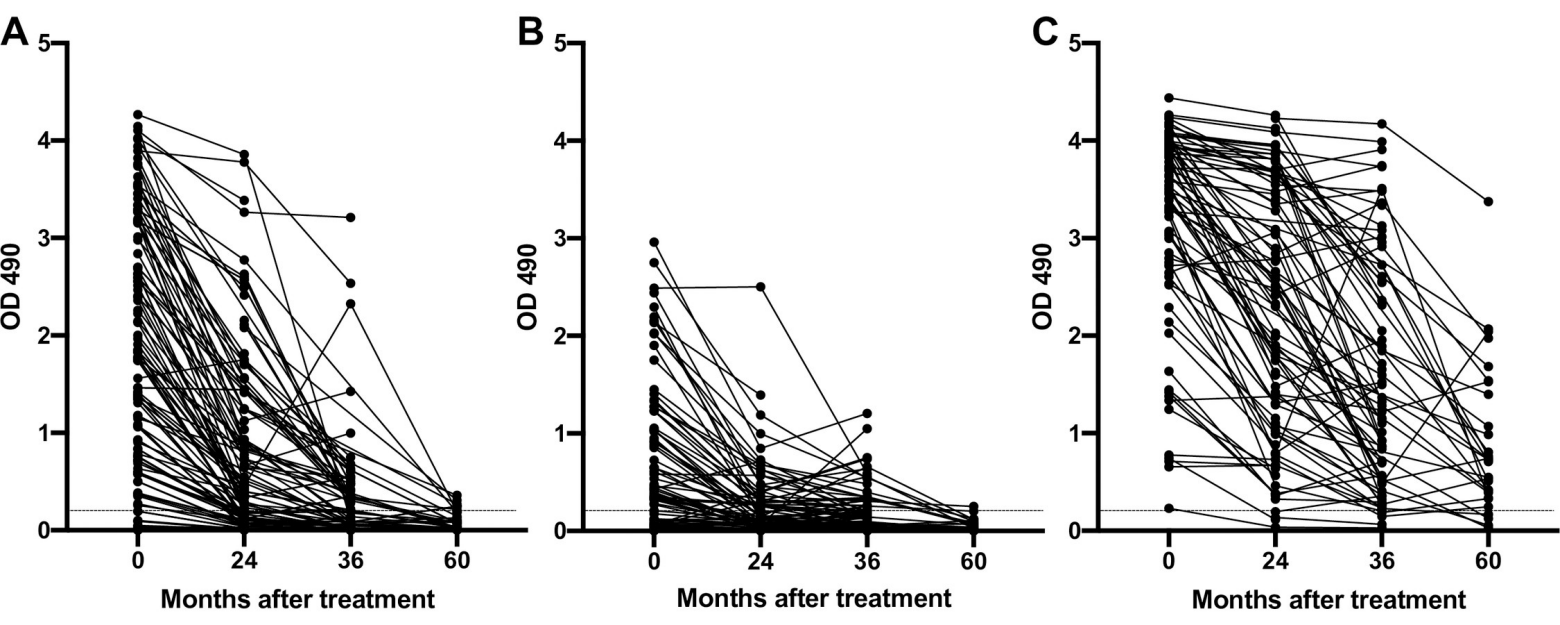
Changes in anti-filarial antibody levels following treatment in a Papua New Guinea treatment trial. Graphs show the OD_490_ values from (A) anti-rWb-Bhp-1, (B) anti-rWb123 and (C) anti-rBm14 IgG_4_ ELISAs before and after treatment (all treatment arms included). The OD_490_ values over time for each individual are connected by black lines. The dotted black line shows the threshold for antibody positivity (OD = 0.2).

Because prolonged positivity after treatment has been reported for several antibody based LF diagnostic tests after treatments that clear Mf, we assessed changes in antibodies to recombinant filarial antigens after treatment with samples from a randomized controlled treatment trial conducted in Papua New Guinea. Participants were treated with one of 3 treatment arms: IDA once; DEC with albendazole once (DA x1); or DEC with albendazole once yearly for 3 years (DA x3) [2]. Sera from patients in this trial were collected pre-treatment and then 24, 36 and 60 months after the first treatment, but only a subset of those in the IDA arm were followed at 60 months [1,2]. Sera were tested for IgG_4_ antibodies to rWb-Bhp-1, as well as rWb123 and rBm14 by ELISA (**Fig 1 and Table 2**). We found that 89/99 (90%) of pretreatment samples had antibodies reactive to rWb-Bhp-1. These 99 pretreatment samples included 23 samples from which we have previously reported anti-rWb-Bhp-1 antibody levels [19]. 55/78 (71%) baseline samples contained antibodies to rWb123 and 77/78 (99%) samples had antibodies to rBm14. We also calculated the percent of participants who had antibodies to either rWb-Bhp-1 or rWb123, to assess the potential of a multiplexed assay (Table 2). This did not substantially increase the pre-treatment sensitivity. Antibodies to all three diagnostic antigens decreased after treatment, but decreases were much more dramatic and rapid for antibodies to Wb-Bhp-1 and Wb123 than to Bm14. As assessed by ELISA OD_490,_ median antibody levels at each time point were significantly different (by Kruskal-Wallis, p<0.001). Furthermore, the percentages of participants with identifiable anti-filarial antibodies decreased over time (**Table 2**).

**Table 2:** Number and percentages of samples with detectible IgG_4_ antibodies to recombinant filarial antigens before and after treatment in a Papua New Guinea treatment trial.

	Number positive / Number tested (%)			
Antigen	0 month	24 month	36 month	60 month
**rWb-Bhp-1**	89/99 (90%)	64/105 (61%)	34/71 (48%)	5/29 (17%)
**rWb123**	55/78 (71%)	31/84 (37%)	24/70 (34%)	2/29 (7%)
**rWb-Bhp-1 + rWb123**	72/78 (92%)	60/84 (71%)	54/70 (77%)	6/29 (21%)
**rBm14**	77/78 (99%)	80/84 (95%)	67/72 (93%)	26/29 (90%)

**Fig 2:**
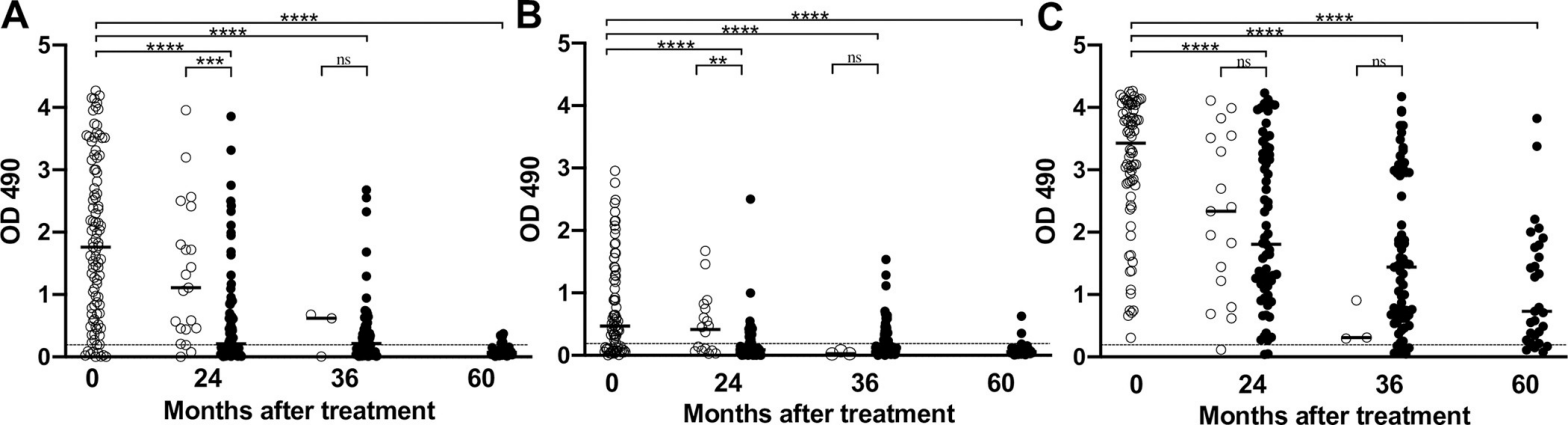
Changes in anti-filarial antibody levels following treatment in a Papua New Guinea treatment trial. Graphs show the OD_490_ for the (A) anti-rWb-Bhp-1, (B) anti-rWb123 and (C) anti-rBm14 IgG_4_ ELISAs before and after treatment, with data stratified by microfilaremia (Mf) status. Open circles indicate persistent Mf, closed circles indicate cleared Mf. Median values are indicated by the black bar. The dotted black line shows the threshold for antibody positivity (OD = 0.2). Significance, as assessed by the Mann-Whitney U test, is indicated (**** is p<0.0001, *** is p<0.001, ** p<0.01, ns is not significant).

The different treatment regimens were variably successful at clearing Mf from the blood by 24 months after treatment, but most participants in all treatment groups were amicrofilaremic by 36 months [2]. When we stratify the data based on the presence of Mf after treatment, those without Mf had significantly lower levels of antibodies to rWb-Bhp-1 and rWb123 by 24 months after treatment (**Fig 2**). There was no significant difference in antibodies to rBm14 at 24 months after treatment between those with and without persistent Mf. There were too few participants with persistent Mf at 36 or 60 months after treatment to meaningfully compare antibody levels in people with and without Mf at those timepoints. Reductions in antibody OD values did not differ by treatment arm (**S1 Fig**), although the study was likely underpowered to detect such differences, and only IDA participants were followed out to 60 months. Furthermore, reductions in antibody OD values did not differ by CFA semi-quantitative score (**S2 Fig**).

**Fig 3:**
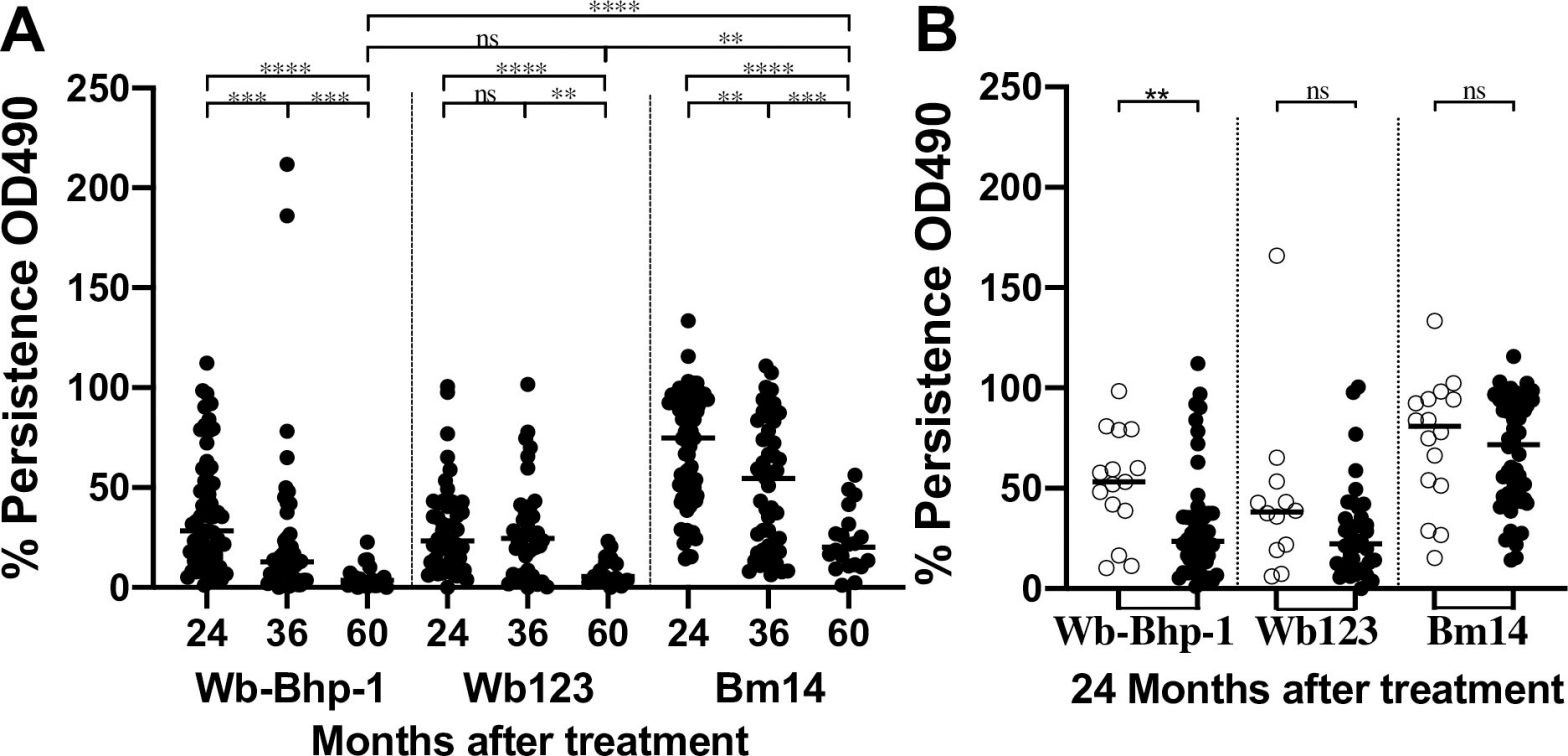
Persistence of anti-filarial antibodies after treatment in a Papua New Guinea treatment trial. Graphs show the persistence, as a percentage of the ELISA OD_490_ values for antibodies to the indicated recombinant antigen relative to the pre-treatment value for each individual, at the indicated time point (A), or with the 24 month follow up data stratified by microfilaremia status (B). In panel B, open circles indicate persistent Mf, closed circles indicate cleared Mf. Median values are indicated by the black bar. Significance as assessed by the Mann-Whitney U test is indicated (**** is p<0.0001, *** is p<0.001, ** p<0.01, ns is not significant).

We also calculated changes in IgG_4_ ELISA OD_490_ for each antigen over time in individual participants. Because of the large spread in OD_490_ values for the pretreatment timepoint, this gave us a better assessment of change over time. While antibodies to all three antigens decreased after treatment, these changes were faster and more complete for antibodies to Wb-Bhp-1 and Wb123 than to Bm14 (**Fig 3A**). Furthermore, when we stratify the data by Mf status, participants who were Mf negative 24 months after treatment had significantly greater decreases in OD values for anti-rWb-Bhp-1 antibodies than those with persistent Mf. In contrast, decreases in OD values in the anti-rWb123 or anti-rBm14 assays at 24 months did not differ based on persistence of Mf (**Fig 3B**).

**Fig 4:**
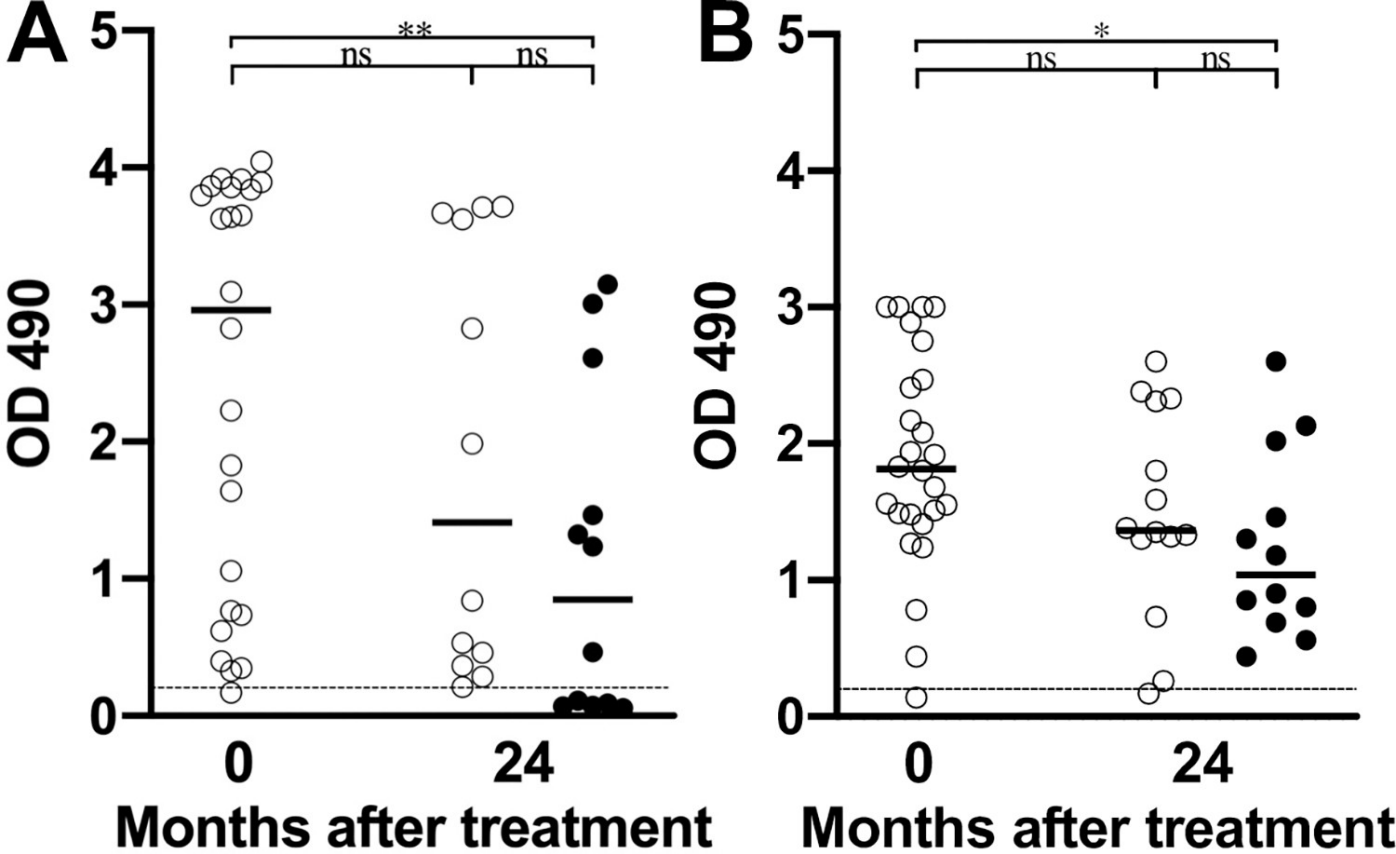
Changes in anti-filarial antibody levels following treatment in a Sri Lanka treatment trial. Graphs show the OD_490_ for the (A) anti-rWb-Bhp-1 or (B) anti-rBm14 IgG_4_ ELISAs before and after treatment initiation, with data stratified by Mf status. Open circles indicate results for samples from people with persistent Mf, and closed circles indicate results from people who cleared Mf. Median values are shown with horizontal black bars. The dotted black line shows the threshold for antibody positivity (OD = 0.2). Significance as assessed by the Mann-Whitney U test is indicated (** is p<0.01, * is p<0.05, ns is not significant).

We have previously reported that the sensitivity of anti-rWb-Bhp-1 ELISA varied according to the country of origin of the samples tested [19]. Because of this variability, we also tested pre- and post-treatment samples from a randomized control treatment trial conducted in Sri Lanka [22]. This trial enrolled 37 patients and treated them with either 1 low dose of ivermectin (control arm), or low dose ivermectin followed by 12 fortnightly full doses of either ivermectin or DEC. The participants were followed for 2 years from the start of the treatment regimen with monitoring of microfilaremia and antigenemia. In this study, 23/24 (96%) of participants had IgG_4_ antibodies reactive to rWb-Bhp-1 before treatment, and 23/24 (96%) of participants had antibodies to rBm14 before treatment, although the single participants without antibodies to these antigens were different. While most participants still had detectible antibodies to both rWb-Bhp-1 and rBm14 by 24 months after treatment, the levels of those antibodies decreased significantly after treatment for both antigens (by Mann Whitney U test, p<0.02) (**Table 3**). However, there was a significant difference in the percent persistence of anti-rWb-Bhp-1 and anti-rBm14 (by Mann Whitney U test, p<0.05), with anti-rWb-Bhp-1 and anti-rBm14 antibodies showing median percent antibody persistence of 44% and 77%, respectively. Furthermore, when we stratify the data by Mf status, only those who cleared microfilaremia had significant decreases in antibody level (**Fig 4**).

**Table 3:** Number and percentages of samples with detectible IgG_4_ antibodies to recombinant filarial antigens before and after treatment in a Sri Lanka treatment trial.

	Number positive / Number tested (%)	
Antigen	0 month	24 month
**rWb-Bhp-1**	23/24 (96%)	19/24 (79%)
**rBm14**	23/24 (96%)	23/24 (96%)

### Anti-rWb-Bhp-1 ELISA results with legacy samples from Egypt and India

**Fig 5:**
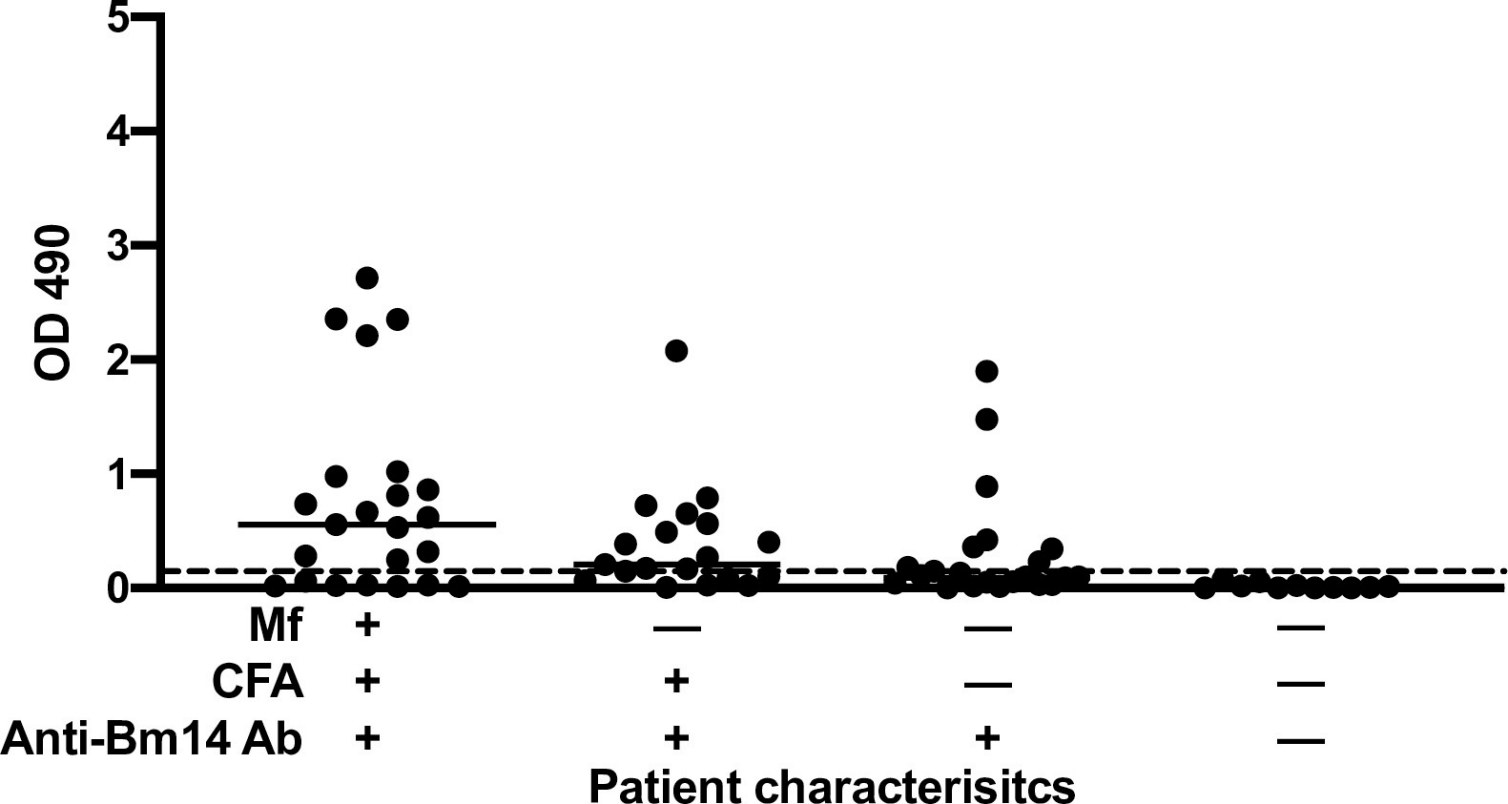
Antibodies to rWb-Bhp-1 in samples from an endemic area in Egypt stratified by results of other tests for lymphatic filariasis. Graph shows the individual OD_490_ values for the anti-rWb-Bhp-1 IgG_4_ ELISA for samples according to the presence of other *W*. *bancrofti* infection markers: microfilaremia (Mf), circulating filarial antigenemia (CFA), and IgG_4_ antibodies to rBm14. Median values are indicated by the black bar. The dotted black line shows the threshold for antibody positivity (OD = 0.2).

For this part of the study, we used samples from people with a range of different LF markers of infection or with clinically evident LF to further characterize the anti-rWb-Bhp-1 IgG_4_ ELISA. We tested sera from a community survey conducted in Egypt prior to their LF elimination campaign in an area with an Mf prevalence of 7.7% (by membrane filtration) and a CFA prevalence of 11.2% [20]. These samples were from people over 9 years of age who were previously assessed for Mf, CFA, and antibodies to rBm14. Results are shown in **Fig 5**: 16 of 22 (73%) of subjects who were positive for Mf, CFA, and anti-rBm14 antibodies had antibodies to rWb-Bhp-1. In contrast, only 10 of 19 (53%) people who were Mf negative but positive for CFA and 7 of 40 (17.5%) endemic normals (Mf and CFA negative) had antibodies to rWb-Bhp-1. All 7 of the positive endemic normal also had anti-rBm14 antibodies. None of 13 (0%) people who were negative for Mf, CFA, and anti-rBm-14 antibodies had antibodies to rWb-Bhp-1 (**Fig 5**). These results suggest that anti-rWb-Bhp-1 antibodies were more closely linked to study participant’s Mf status than to their CFA status. They also suggest that most uninfected people living in LF endemic areas lack antibodies to rWb-Bhp-1.

Anti-filarial antibody tests often have lower sensitivity in people with clinical LF [25,26]. We tested 16 legacy plasma samples from people in southern India with clinical LF (lymphedema, recurrent lymphadenitis and/or hydrocele), three of whom were microfilaremic [21]. We tested these sample for antibodies to rWb-Bhp-1, rWb123 and rBm14 (**S3 Fig**). Only 2 of 16 (12.5%) samples had detectible anti-rWb-Bhp-1 antibodies, and those 2 samples also had antibodies to rWb123 and rBm14 (**S3 Fig**). These samples were from people with Mf; one had a night blood Mf count of 6 per 20ul smear, and the other had a count of >500 Mf/ml by membrane filtration. The third microfilaremic person had a Mf count of 2 per 20ul blood smear, and their serum did not have detectable antibodies to any of the tested recombinant filarial antigens. Samples from 5 of 16 (31%) individuals with clinical LF had low levels of anti-rBm14 antibodies but no detectible antibodies to rWb-Bhp-1 or rWb123 (**S3 Fig**). All 5 of these individuals were amicrofilaremic.

### Wb-Bhp-1 was not detected in serum samples by sandwich ELISA

We also tested whether Wb-Bhp-1 protein could be detected in serum from LF patients as an antigen biomarker. We developed a sandwich ELISA using both mouse and rabbit polyclonal antibodies to rWb-Bhp-1 that could detect 5ng/ml of purified rWb-Bhp-1. We also found that the rWb-Bhp-1 retained immuno-reactivity after heating to 70°C in EDTA, a treatment that can release antigens from immune complexes [27]. However, we were unable to detect Wb-Bhp-1 protein in sera from 12 microfilaremic individuals with this assay (**S4 Fig**). Therefore, while detection of antibodies to rWb-Bhp-1 is useful for diagnosis, Wb-Bhp-1 is not a reliable biomarker for infection with our sandwich ELISA.

## Discussion

Recent studies have shown that IDA is highly effective for clearance of Mf in individuals and in populations. Indeed, 5 years after a single dose of IDA, 75% of participants in a treatment trial performed in Papua New Guinea had persistent CFA, while only 3% had Mf, either due to persistence or reinfection [1]. This highlights the difficulty of using CFA test results alone for informing decisions regarding when to stop MDA in an area. Antibody-based LF diagnostics are attractive for a variety of reasons: day time blood samples can be utilized, tests can be formatted as inexpensive point of care assays, and anti-filarial antibodies often can be detected before the onset of Mf or CFA. However, antibodies to some filarial antigens remain detectable for years after effective treatment, and this limits the value of antibody tests for post-MDA surveillance. Antibody tests that are closely correlated with Mf status and that clear more rapidly than CFA after effective treatment could be useful for MDA stopping decisions and for post-MDA surveillance.

We have previously reported that an ELISA for IgG_4_ antibodies to rWb-Bhp-1 has good sensitivity with samples from people with *W*. *bancrofti* Mf, although the sensitivity varies by geographic area [19]. Here, we have assessed the impact of anti-filarial treatment on antibodies to recombinant filarial antigens Wb-Bhp-1, Wb123, and Bm14 with samples from a clinical trial conducted in Papua New Guinea. While the anti-rWb-Bhp-1 ELISA was more sensitive than the anti-rWb123 ELISA, it was less sensitive than the anti-rBm14 ELISA. However, we demonstrate that anti-rBm14 antibodies persist long after treatment, with 90% of participants tested retaining detectible levels by 60 months after treatment. While less sensitive in pre-treatment samples, IgG_4_ antibodies to rWb-Bhp-1 and rWb123 cleared more rapidly after treatment than IgG_4_ antibodies to rBm14, with only 17% and 7% of samples positive, respectively, 60 months after treatment. Similarly, IgG_4_ antibodies to rWb-Bhp-1 decreased more after treatment than antibodies to rBm14 in samples from a clinical trial in Sri Lanka.

We also found evidence that, after treatment, anti-rWb-Bhp-1 antibodies are more closely correlated with microfilaremia status than other markers of LF infection such as CFA. More people who were microfilaremic and antigenemic had anti-rWb-Bhp-1 antibodies than those who were antigenemic alone. Furthermore, those with persistent Mf after treatment had higher levels of anti-rWb-Bhp-1 antibodies than those who had cleared Mf.

Previous work has demonstrated that people with clinical LF manifestations such as lymphedema or hydrocele are often amicrofilaremic, with <1 to 5% microfilaremia rates [28–30]. It has also been reported that these patients have higher levels of circulating immune complexes, but lower levels of anti-filarial antibodies compared with microfilaremic individuals [25,26,31]. Our study found that anti-rWb-Bhp-1 antibodies are not sensitive for the diagnosis of clinical LF. As most people with clinical LF are amicrofilaremic, this is further evidence that anti-rWb-Bhp-1 antibodies are correlated with the presence of microfilaremia. This correlation is interesting given what is known about the expression of Wb-Bhp-1. This antigen is a *W*. *bancrofti* homologue of BmR1, which is present in microfilariae based on immunohistochemistry of *B*. *malayi* female worms [19]. Gene expression and proteomic studies have also shown that it is preferentially expressed by Mf [32–36]. Therefore, it is unsurprising that anti-rWb-Bhp-1 antibodies are associated with microfilaremia. In contrast to Wb-Bhp-1, Bm14 and Wb123 are not specifically linked to Mf. Bm14 is expressed by several *B*. *malayi* stages [33,34]. Wb123 was identified based on high expression in the L3 stage of *W*. *bancrofti* [10], but its homologue in *B*. *malayi* is now known to be expressed by other parasite stages as well [33,34]. While anti-rWb123 antibody levels were significantly lower in those who cleared Mf by 24 months, there was no significant difference in percent persistence of anti-rWb123 antibodies at 24 months based on that individual’s Mf status. However, there was a significant difference in percent persistence of anti-rWb-Bhp-1 antibodies at 24 months based on that individual’s Mf status. Therefore, post-treatment anti-rWb123 antibodies did not correspond as well to Mf status as antibodies to rWb-Bhp-1.

There were several limitations to this study. The study of samples from the PNG clinical trial was limited by the fact that we only had samples from a subset of participants collected 60 months after treatment and that all of those subjects were treated with IDA. This limited our ability to detect differences in the impact of treatment regimen on antibody persistence. The fact that there were few Mf-positive participants at later follow-up times in that trial limited our ability to compare persistence of antibodies in persons with and without persistent Mf after treatment. Another limitation of the study is that it only tested samples from treatment trials performed in PNG and Sri Lanka, where prior studies have demonstrated high sensitivity for the rWb-Bhp-1 ELISA. The assay would not be expected to work as well with samples from areas where the antibody test has been shown to have low sensitivity with samples from infected persons prior to treatment.

In conclusion, our results suggest that tests for antibodies to rWb-Bhp-1 and rWb123 might be superior to CFA as a tool for post-MDA surveillance, and this may be especially true in areas where Mf prevalence is rapidly reduced following high-coverage MDA with IDA. Antibodies to rWb-Bhp-1 were more commonly present than antibodies to rWb123 prior to treatment, and people with persistent Mf after treatment were also more likely to have persistent anti-rWb-Bhp-1 antibodies. Thus, Wb-Bhp-1 is a promising diagnostic antigen that may prove useful for MDA stopping decisions and for post-MDA surveillance in the Global Programme to Eliminate Lymphatic Filariasis.

## Supporting information

S1 Fig Post-treatment changes in anti-filarial antibody levels according to treatment regimen. Graphs show the ELISA OD_490_ for the (A) anti-rWb-Bhp-1, (B) anti-rWb123 and (C) anti-rBm14 IgG_4_ ELISA before and after treatment, with data stratified by type of treatment, as specified in the legend. Median values are indicated by the black bar. The dotted black line shows the threshold for antibody positivity (OD = 0.2)
(TIF)

S2 Fig Post-treatment changes in anti-filarial antibody levels according to semi-quantitative circulating filarial antigen level. Graphs show the ELISA OD_490_ for the (A) anti-rWb-Bhp-1, (B) anti-rWb123 and (C) anti-rBm14 IgG_4_ ELISA before and after treatment, with data stratified by semi-quantitative Filariasis Test Strip score (0–3), as specified in the legend. Median values are indicated by the black bar. The dotted black line shows the threshold for antibody positivity (OD = 0.2).
(TIF)

S3 Fig Antibodies to rWb-Bhp-1, rWb123 and rBm14 in chronic LF patients from India. Graph shows the individual OD_490_ for the anti-rWb-Bhp-1, anti-rWb123 and anti-rBm14 IgG_4_ ELISA. The dotted black line shows the threshold for antibody positivity (OD = 0.2).
(TIF)

S4 Fig Wb-Bhp-1 antigen was not detected in patient sera. Graph demonstrates results from a Wb-Bhp-1 sandwich ELISA on sera samples from 12 microfilaremic individuals (2 from Sri Lanka (SL), and 5 each from Papua New Guinea (PNG) and Cote d’Ivoire (CDI) (as listed in Table 1), or 50ng Wb-Bhp-1 as a positive control (+) or buffer control (-).
(TIF)
