# Supplementary figures and images for: IgG4 antibodies to the recombinant filarial antigen Wb-Bhp-1 decrease dramatically following treatment of lymphatic filariasis
Source: PLoS Negl Trop Dis. 2023 Jun 7;17(6):e0011364. doi: 10.1371/journal.pntd.0011364 (PMC10246830; doi:10.1371/journal.pntd.0011364)

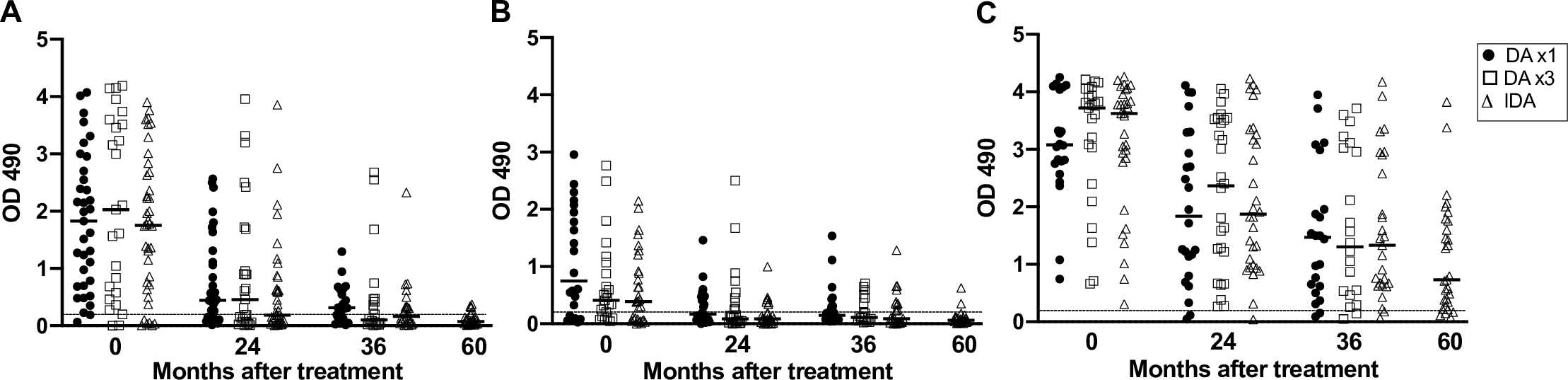

Supplement: S1 Fig — Graphs show the ELISA OD490 for the (A) anti-rWb-Bhp-1, (B) anti-rWb123 and (C) anti-rBm14 IgG4 ELISA before and after treatment, with data stratified by type of treatment, as specified in the legend. Median values are indicated by the black bar. The dotted black line shows the threshold for antibody positivity (OD = 0.2) (TIF) [file pntd.0011364.s001.tif]

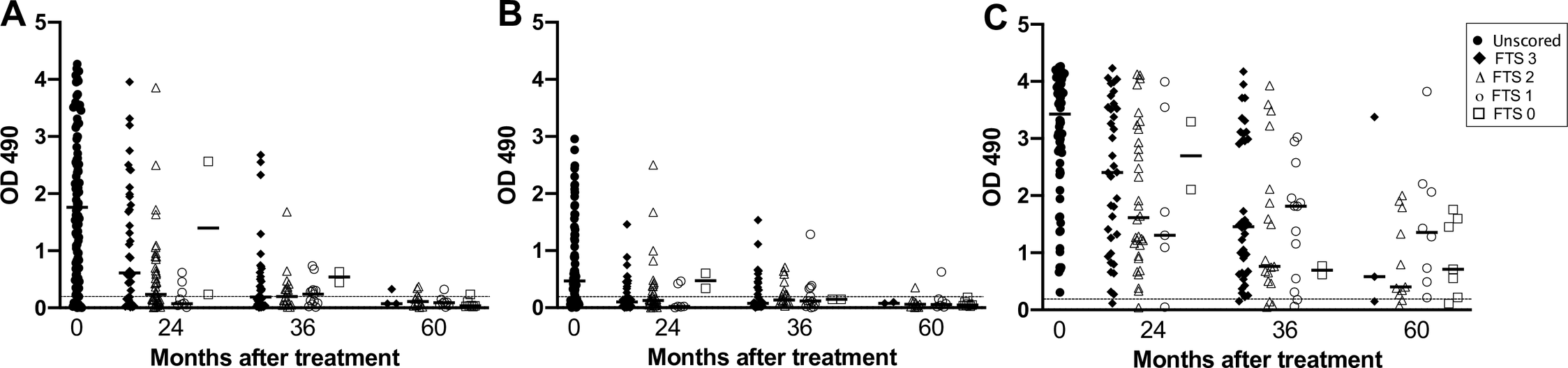

Supplement: S2 Fig — Graphs show the ELISA OD490 for the (A) anti-rWb-Bhp-1, (B) anti-rWb123 and (C) anti-rBm14 IgG4 ELISA before and after treatment, with data stratified by semi-quantitative Filariasis Test Strip score (0–3), as specified in the legend. Median values are indicated by the black bar. The dotted black line shows the threshold for antibody positivity (OD = 0.2). (TIF) [file pntd.0011364.s002.tif]

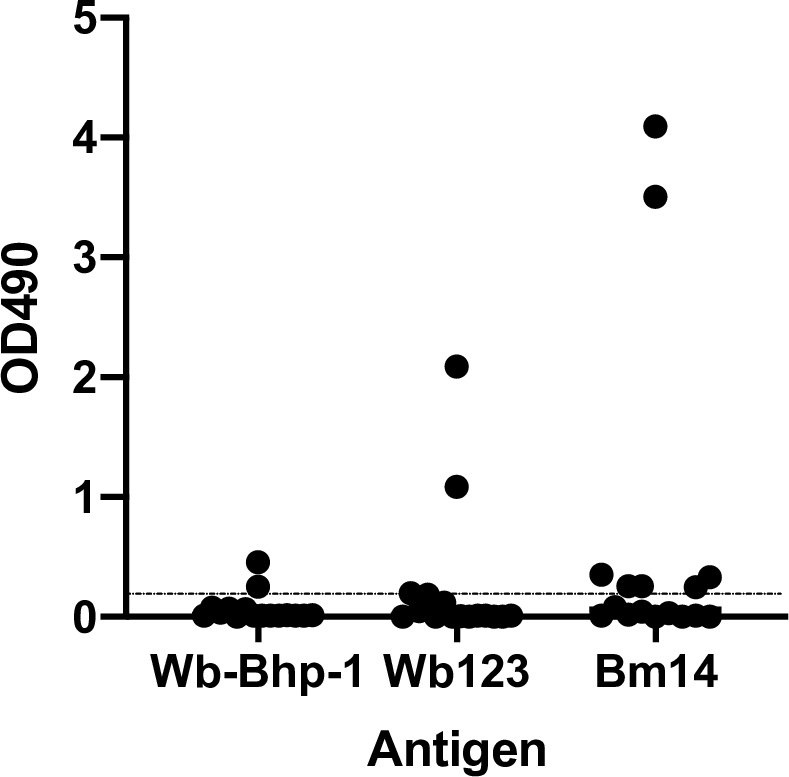

Supplement: S3 Fig — Graph shows the individual OD490 for the anti-rWb-Bhp-1, anti-rWb123 and anti-rBm14 IgG4 ELISA. The dotted black line shows the threshold for antibody positivity (OD = 0.2). (TIF) [file pntd.0011364.s003.tif]

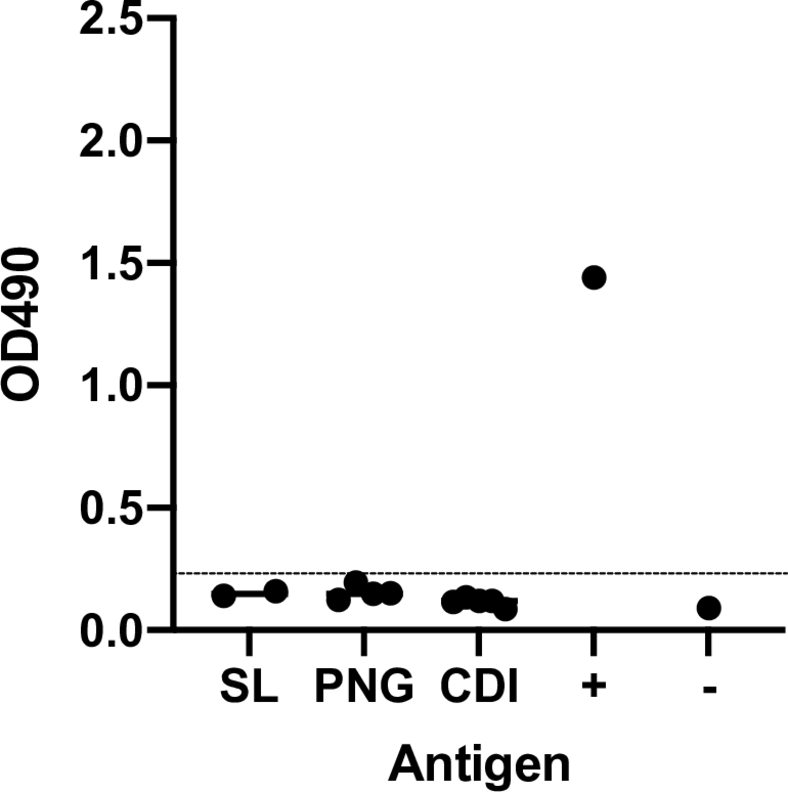

Supplement: S4 Fig — Graph demonstrates results from a Wb-Bhp-1 sandwich ELISA on sera samples from 12 microfilaremic individuals (2 from Sri Lanka (SL), and 5 each from Papua New Guinea (PNG) and Cote d’Ivoire (CDI) (as listed in Table 1), or 50ng Wb-Bhp-1 as a positive control (+) or buffer control (-). (TIF) [file pntd.0011364.s004.tif]
